# Optical Fiber Sensing Based on Reflection Laser Spectroscopy

**DOI:** 10.3390/s100301823

**Published:** 2010-03-05

**Authors:** Gianluca Gagliardi, Mario Salza, Pietro Ferraro, Edmond Chehura, Ralph P. Tatam, Tarun K. Gangopadhyay, Nicholas Ballard, Daniel Paz-Soldan, Jack A. Barnes, Hans-Peter Loock, Timothy T.-Y. Lam, Jong H. Chow, Paolo De Natale

**Affiliations:** 1 Consiglio Nazionale delle Ricerche, Istituto Nazionale di Ottica (INO), Via Campi Flegrei, 34 I-80078 Napoli, Italy; E-Mails: mario.salza@ino.it (M.S.); pietro.ferraro@ino.it (P.F.); paolo.denatale@ino.it (P.D.N.); 2 Engineering Photonics Group, School of Engineering, Cranfield University, Bedford MK43 OAL, UK; E-Mails: e.chehura@cranfield.ac.uk (E.C.); r.p.tatam@cranfield.ac.uk (R.P.T.); 3 Central Glass & Ceramic Research Institute (CSIR), 196, Raja S.C.Mullick Road, Kolkata-700 032, India; E-Mail: tkg@cgcri.res.in (T.K.G.); 4 Department of Chemistry, Queen’s University, Kingston, ON, K7L 3N6, Canada; E-Mails: 5ngb@queensu.ca (N.B.); danielps@rogers.com (D.P.-S.); jbarnes@chem.queensu.ca (J.A.B.); hploock@chem.queensu.ca (H.-P.L.); 5 Centre for Gravitational Physics, Research School of Physics and Engineering, Australian National University, Canberra, ACT 0200, Australia; E-Mails: timothy.lam@anu.edu.au (T.T.-Y.L.); jong.chow@anu.edu.au (J.H.C.)

**Keywords:** Fiber Bragg gratings, fiber resonator, Pound-Drever-Hall method, frequency locking, laser-frequency modulation, high-birefringence fiber

## Abstract

An overview on high-resolution and fast interrogation of optical-fiber sensors relying on laser reflection spectroscopy is given. Fiber Bragg-gratings (FBGs) and FBG resonators built in fibers of different types are used for strain, temperature and acceleration measurements using heterodyne-detection and optical frequency-locking techniques. Silica fiber-ring cavities are used for chemical sensing based on evanescent-wave spectroscopy. Various arrangements for signal recovery and noise reduction, as an extension of most typical spectroscopic techniques, are illustrated and results on detection performances are presented.

## 1. Introduction

Optical fiber systems have made great impact in the field of sensing thanks to the growth of the optoelectronics and fiber-optic communication industries. Many of the components used in these markets were devised benefiting from outstanding technologies in the design of materials, optical fibers and light sources. The inherent advantages of fiber optic sensors include their light weight, low cost, small size and ruggedness, making it possible to directly install and integrate them in environments with access difficulty. The immunity to electromagnetic interference, high bandwidth and response are crucial for high sensitivity and high accuracy sensing. The past 20 years have witnessed an intensive research effort on the use of optical fiber sensors to measure different physical and chemical parameters [1,2].

Among the optical sensors, FBGs have been demonstrated to be very promising as mechanical probes for a number of applications. Several interrogation systems have been developed so far, often based on broad-emission radiation sources in conjunction with either optical spectrum analyzers or filters [3,4]. More recently, sophisticated schemes based on narrow-band laser sources and laser-frequency stabilization methods were devised, achieving strain sensitivity at the 10^−12^ level and better for quasi-static and dynamic monitoring [5–7]. Among them, a significant contribution came from the use of FBG-based resonant structures whose highly-dispersive power near resonance is exploited to measure sub-pm length perturbations over a wide range of frequencies. On the other hand, optical resonators based on high-reflectivity FBGs, fiber loops and silica microspheres have been employed for refractive index and gas sensing. Cavity-enhanced and ring-down techniques enable the extraction of information on light-matter interaction in direct or evanescent-wave spectroscopy schemes [8–10].

## 2. Strain and Temperature Sensing

### 2.1. Bragg Gratings in Highly-Birefringent (HiBi) Fibers

Despite the sensitivity achievable using systems based on laser-spectroscopy techniques or fiber interferometers [6,11,12], a quantitative measurement at very-low frequency (approaching DC) is affected by temperature cross-sensitivity that may limit the ultimate measurement accuracy. This is particularly relevant for field applications where a continuous and reliable operation is required outdoor. A number of solutions which have been proposed to avoid such limitation include the use of reference sensors, two-wavelengths FBGs, chirped gratings and birefringent fibers, but often with serious degradation of the measurement precision [13]. Here, we combine the advantages of laser-spectroscopic methods, which improve detection sensitivity, with the use of FBG sensors fabricated in HiBi (Panda) fibers to simultaneously monitor temperature and strain [14]. We describe the proposed methods, the experimental set-ups and the preliminary results obtained by a single FBG and an in-fiber Fabry-Pérot (FFP) cavity.

**Figure 1.**
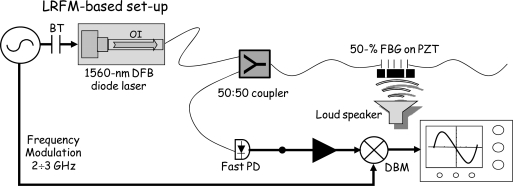
RF-modulation-based FBG interrogation set-up. PD: photodiode; DBM: double-balanced mixer; BT: bias-tee.

In a previous paper, we reported on the implementation of a strain sensing apparatus based on a frequency-modulated diode-laser at 1,560 nm, which was aimed at the static and dynamic interrogation of single FBGs with improved sensitivity [15]. Our main concern was to achieve a quantitative understanding of all aspects that influence the performance of such systems. This procedure was essential as it enabled further optimization and improvement in the interrogation system, in order to develop a portable FBG-based sensor with capabilities that are necessary for field use. Figure 1 illustrates the basic interrogation scheme. The system relied on radio-frequency (RF) sideband generation on the laser beam, via current modulation, and heterodyne detection of the FBG reflected light. If the sideband frequency is high enough compared to the FBG width, its reflection spectrum can be treated as a molecular absorption line. Demodulation at that frequency is performed by a double-balanced mixer which yields a highly-dispersive signal with a zero-crossing around the Bragg’s resonance in quiescent conditions. Thus any mechanical strain on the sensor will be converted into a non-zero voltage output. This deviation can be employed as a discriminator (error) for Pound-Drever-Hall (PDH) [16] frequency locking of the laser onto the Bragg grating’s peak for continuous tracking of the sensor.

**Figure 2.**
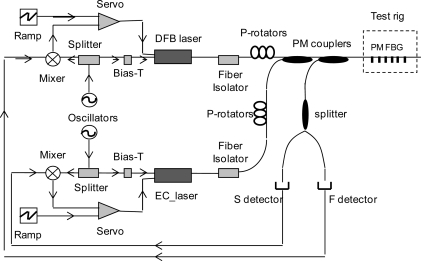
Experimental set-up: P-rotators: polarization rotators; splitter: polarization splitter; EC laser: extended cavity laser.

**Figure 3.**
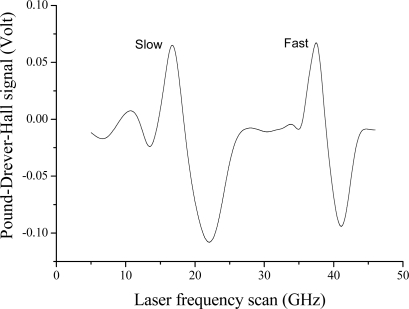
Mixer output line shapes over a laser-frequency scan around the PM Bragg resonances with a 45° linear polarization state.

A slightly modified scheme has been applied to a FBG with 50% peak reflectivity that was fabricated in a polarization-maintaining (PM) fiber with Panda configuration. This is shown in Figure 2. A distributed feedback diode laser (DFBL) and an extended-cavity diode laser (ECL), both emitting around 1,560 nm, were equipped with Panda-fiber pig-tails. The lasers were driven by low-noise current and temperature controllers, and their linear states of polarization (SOP) were aligned with the fiber’s slow and fast axes, respectively. Sidebands were superimposed on the laser carrier frequencies by applying signals in the GHz range at their bias-tee inputs. DFBL was modulated at *f_1_* = 2 GHz while ECL was modulated at *f_2_* = 1 GHz and both were coupled to the FBG through a 3 dB PM fiber coupler. The HiBi-grating reflected signal presented two distinct peaks corresponding to slow and fast SOPs, which were separated by a polarization splitter and detected by two different photodiodes. A double-balanced mixer (DBM) demodulated the detector voltages at frequency *f_1,2_* and yielded dispersive-like signals resulting from the phase shift between sideband-carrier beats [7]. Since the FBG full-width at half maximum (FWHM) was about 7 GHz, a 1–2 GHz modulation was able to generate a suitable error signal. When each laser was resonant with the corresponding FBG peak, the mixers provided a voltage crossing zero in correspondence to the maximum reflectivity. DFBL and ECL gave rise to mixer signals centered at slightly different wavelengths as a consequence of the Panda fiber polarization-mode dispersion (∼0.16 nm). An example is in Figure 3. These signals were directed to proportional-integrative (PI) electronic loops (acting as servos), which control the laser current and the extended-cavity length of the ECL. By active locking of each laser on the FBG peaks, the correction signals serve as real-time monitors of strain over a wide dynamic range.

In our set-up, we could apply longitudinal strain (*ε_L_*) to the grating by a piezo-electric transducer (PZT) that stretched the fiber attached to it, and transverse strain (*ε_T_*) by pressing on the FBG along the slow axis of the fiber by a specially-designed transverse loading machine [17]. The latter allowed exerting an evenly-distributed pressure between two glass surfaces in contact with the fiber. The actual deformations could be readily calibrated using a reference FBG made in a single mode fiber, whose gauge factor was preliminarily determined by applying known weights. The single-mode FBG was then attached vertically to the transverse load applied to the PM FBG resulting into axial deformation of the single-mode fiber. Temperature variations (Δ*T*) could also be imposed on the fiber by means of a Peltier element in thermal contact with the FBG. Here, the temperature was controlled by a proportional-integrative-derivative (PID) stabilizer (resolution ≈ 0.01 °C) and measured by an AD 590 probe. Once the lasers were locked to the reflection peaks, for a given thermal or mechanical perturbation, we analyzed the corresponding changes in the servo feedback signal and retrieved the response of the PM FBG to these measurands. By comparing all calibrations to the correction signal voltage, we obtained the response factors of the system to *ε_L_*, *ε_T_* and Δ*T* as a function of the output voltages, *i.e.*, the locking feedback signals of the two crossed-polarized lasers.

**Figure 4.**
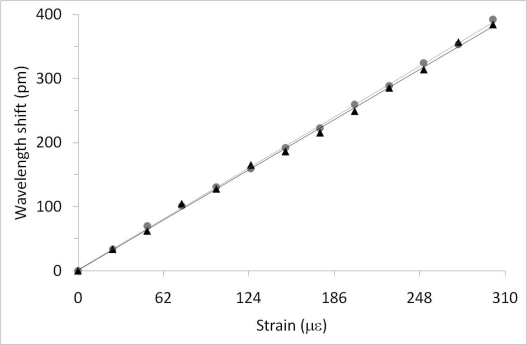
Longitudinal strain response for PM FBG in both the Fast and Slow axes of the fiber (1.264 ± 0.02 pm/με and 1.282 ± 0.009 pm/με for slow and fast axes respectively). 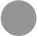 (grey), Fast and ▴ (black), Slow axes.

**Figure 5.**
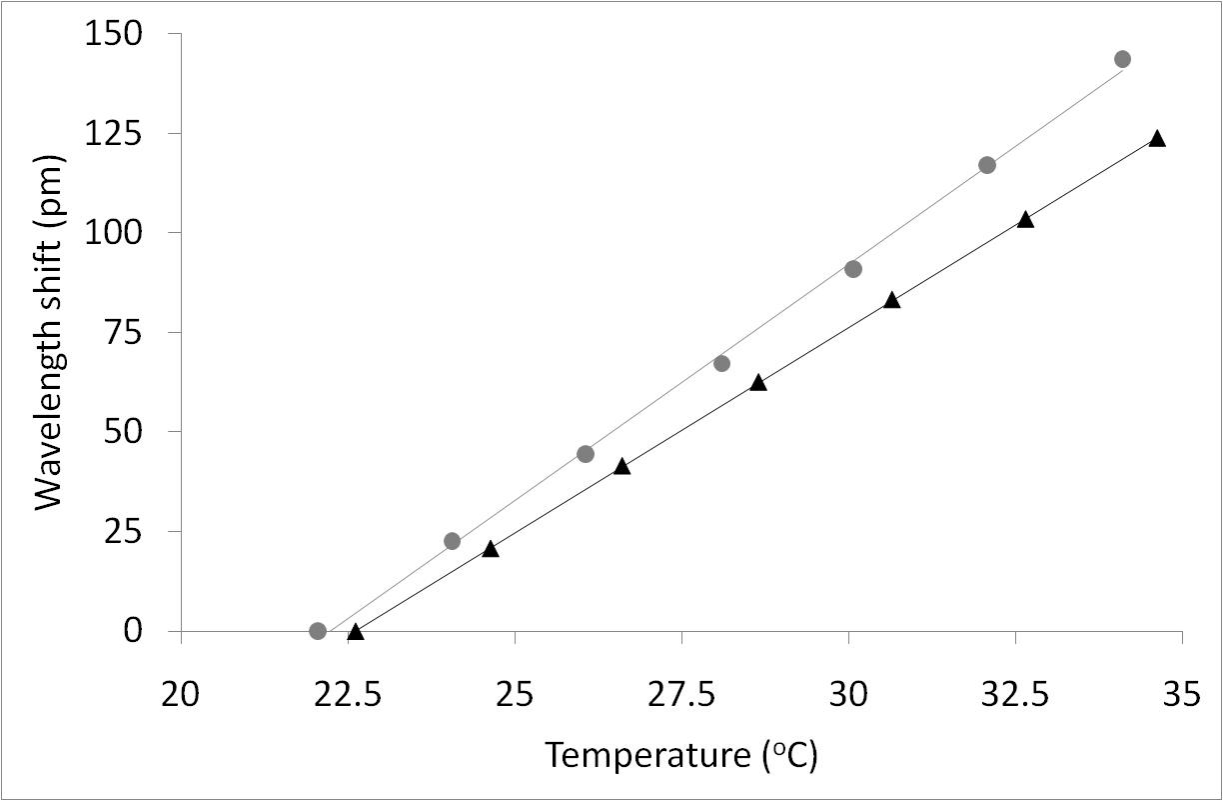
Temperature response for PM FBG in both the Fast and Slow axes of the fiber (10.326 ± 0.02 pm/°C and 11.858 ± 0.2 pm/°C for slow and fast axes respectively). 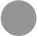 (grey), Fast and ▴ (black), Slow axes.

Calibration measurements with the single sensor have been carried out applying longitudinal loads using the PZT in a free-running configuration. In Figure 4, linear fits to the graphs give longitudinal strain responses of 1.26 ± 0.02 pm/με and 1.282 ± 0.009 pm/με for the slow and fast axes, respectively. Temperature factors obtained for the same FBG were 10.326 ± 0.02 pm/°C and 11.858 ± 0.2 pm/°C for the slow and fast axes respectively (Figure 5). The results for longitudinal strain sensitivity of the HiBi FBG are barely distinguishable while temperatures are significantly different. Noise effects that limit measurement accuracy, e.g., spurious optical reflections, can be significantly reduced by using optical components with better anti-reflection coatings in the polarization analyzer. Application experiments for this technique to the measurement of strain (longitudinal) and temperature, in both dynamic and static modes, have been performed.

**Figure 6.**
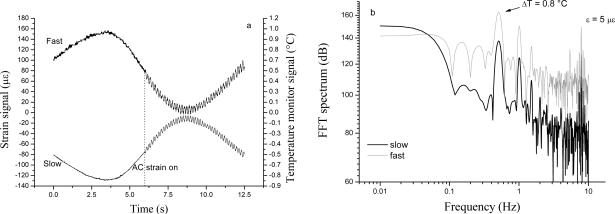
(a) Time response of the laser-locked system when a sine voltage is applied to the PZT (8 Hz) attached to the FBG and a periodic temperature change created with the PID controller (0.5 Hz). (b) FFT spectrum of Figure 6a (50 mHz resolution bandwidth). The traces were shifted by 30 dB for sake of clarity.

As a proof of efficiency of the sensing method, simultaneous detection of AC longitudinal strain (5 με peak-to-peak) and temperature change (0.8 °C peak-to-peak) is demonstrated in Figure 6. It is worth noting, from Figure 6a, how the system detects both strain and temperature variations applied at different frequencies simultaneously by the slow-axis (ECL) and the fast-axis (DFBL) signals. These signals are currently not corrected for the difference between the two laser servo gains: indeed they have opposite sign since the locking correction for the fast SOP is fed back to the injection current of the DFB laser, while a PZT is used for low-frequency control of the extended-cavity laser for the fast SOP. The Fourier transform of Figure 6a, shown in Figure 6b, has two marked peaks above the background, corresponding to mechanical (8 Hz) and thermal (0.5 Hz) stress excited in the fiber. A preliminary sensitivity estimate, based on the noise floor of Figure 6b, indicates that quasi-static strain near to the 10 nε_rms_/√Hz level is within the capability of our system. At higher acoustic frequencies (500—1000 Hz), we measured a sensitivity improvement of nearly a factor of 100 (100 pε/√Hz). Analogously, for small temperature changes, a minimum detectable temperature variation of 5 mK_rms_/√Hz can be extrapolated.

Strain and temperature variations can be thus detected simultaneously by a single sensor. An experimental test on cross-correlation between transverse strain and longitudinal strain in a dynamic measurement regime will be the subject of future investigations.

### 2.2. Fiber Bragg-Grating Resonators

A different kind of apparatus was developed with high-finesse in-fiber Bragg-grating Fabry-Pérot (FFP) resonators as strain sensors. The resonator is formed by two high-reflectivity single-mode FBGs at a relative distance of 100 mm. Small optical pathlength variations in the intra-cavity fiber are turned into frequency shift of the narrow resonance. A first demonstration of its sensitivity to strain signals is given below. Similarly to section 2.1, a diode laser is actively locked to the resonator by an optical-electronic loop.

**Figure 7.**
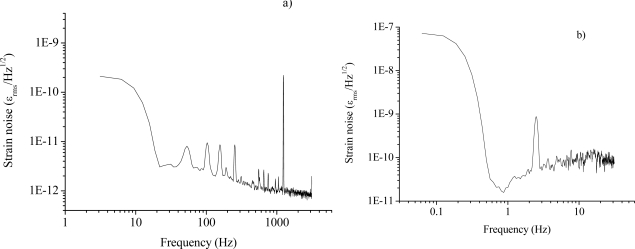
Noise spectral density of the FBG-resonator locking signal for different excitation frequencies in the SM-fiber cavity: (a) a sharp peak is evident at 1.2 kHz with a noise increase towards low frequencies and spurious oscillations due to harmonics of the AC line frequency; (b) the system is capable of detecting deformations down to 2.4 Hz.

Although the interrogating laser can be frequency controlled again by PDH method [7], a different scheme, based on polarization-spectroscopy (PS), was devised. The PS technique was an extension of the well-known Hansch-Couillaud scheme [18] and relied on the birefringence induced by FBG fabrication in the resonator [19,20]. The error signal was obtained by adjusting the SOP of the laser beam at 45° to the fiber birefringence axis and analyzing the cavity-reflected field with a polarization analyzer [21]. In this way, an excellent performance was obtained without using any RF laser modulation or sophisticated electronics. At the same time, a big improvement was obtained by replacing the DFB diode with a narrow-linewidth extended-cavity diode laser. This facilitated the interrogation of the FFP resonances and reduced the noise content of the in-loop signal, deriving from conversion of laser-frequency noise into amplitude noise by the cavity. As for PDH, the strain information is carried by the servo output signal within the locking bandwidth (40 kHz). Signals at different frequencies were applied to the intra-cavity fiber by a PZT (∼1 nε peak-to-peak strain) attached to it and the locking correction signal was analyzed by FFT. Figure 7 shows the sensitivity performance for the PS system, pointing out the strain noise level and the frequency response. The minimum detectable strain level was found to be as low as 1 pε/√Hz, around 1 kHz, and about 40 pε/√Hz around 1–2 Hz [21]. Work is now in progress to further reduce the effect of free-running laser jitter via pre-stabilization on an optical frequency reference.

**Figure 8.**
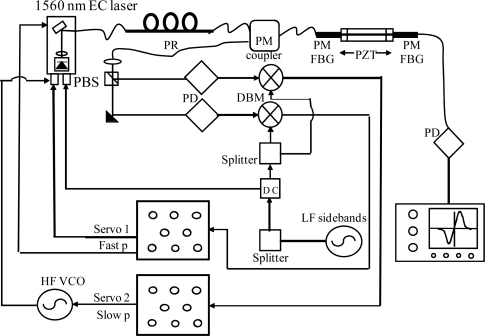
Pound-Drever-Hall interrogation set-up of the HiBi FBG cavity. PD: photodiode; DBM: double-balanced mixer; LF: low-frequency; HF VCO: high-frequency voltage-controlled oscillator; PBS: polarizing cube beamsplitter.

**Figure 9.**
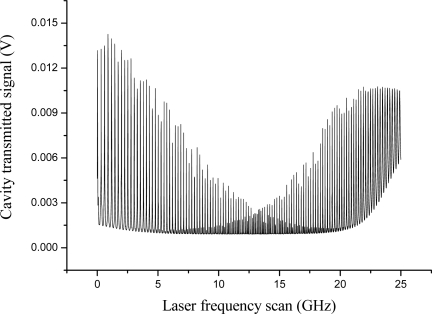
Transmission of the PM FBG resonator for a wide laser sweep (∼0.2 nm).

**Figure 10.**
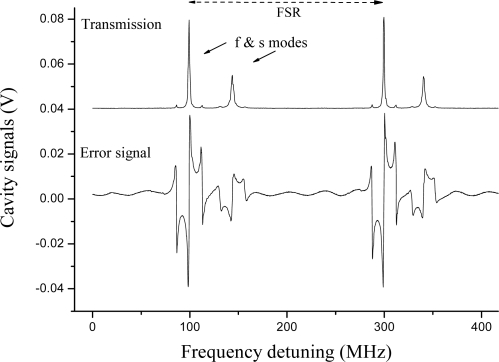
Narrow laser frequency scan equivalent to about one cavity free spectral range (FSR) with RF sidebands at 12 MHz (upper graph). Two peaks appear well separated in frequency by 50 MHz. The PDH signals are also recorded for both polarization eigenmodes.

A PDH scheme was adopted for interrogation of a PM fiber Fabry-Pérot (FFP) cavity. The instrumental arrangement is shown in Figure 8, where the FBG sensor has been replaced by an in-fiber resonator and only one laser is employed. Two high-reflectivity Panda FBGs (reflectivity ∼99 %) were coupled to form a Fabry-Pérot resonator. The Bragg mirrors were separated by about 50 cm and enclosed in aluminum cylinders to keep them mechanically isolated. The cavity had a measured finesse of 300. Again the sensitive element was represented by the intra-cavity fiber and thus small strain signals were excited by a PZT placed between the cylinders. In Figure 9, the cavity transmission was recorded when the laser was swept over a large wavelength range. The narrowest resonances, corresponding to the maximum FBG reflectivity, yielded the minimum transmission level as a consequence of internal fiber losses. It is worth noting that two dispersed mode sequences are present, apparently belonging to different resonators. They have their maximum finesse window centered at different wavelengths, as a consequence of the polarization-mode dispersion in HiBi FBG reflectors. Furthermore, the internal fiber birefringence removes the usual polarization degeneracy of Fabry-Pérot resonance. Each cavity mode is split into two if the incident SOP is not perfectly oriented along one of the principal axes, *i.e*., each resonance consists of a “slow” and a “fast” peak. This is clearly visible in Figure 10 (upper graph). As in the scheme that was described for PM FBGs, a single laser could be frequency-locked to either of the crossed-polarized peaks using different PDH paths so as to independently interrogate each eigenmode at the same time. Firstly, low-frequency (LF) sidebands at 12 MHz were superimposed to the laser beam for frequency locking. Then another pair of sidebands, with relatively strong modulation depth, are generated at about 50 MHz by a tunable voltage-controlled oscillator (HFVCO). One of them played the role of ‘secondary’ carrier to interrogate one of the resonances by frequency locking to it. Reflected light from the cavity was collected by a fiber coupler connected to a polarization analyzer (PBS) to discriminate between fast and slow eigenmodes. The corresponding beat signals at the LF frequency from the mixers were fed back to the laser and the HFVCO by the PI servo for locking the two ‘carriers’ on the corresponding peaks.

Static and dynamic deformations as well as thermal effects on the fiber result in changes of the optical pathlength and thus frequency shifts of the resonances, but with different responses along the principal axes. Strain-induced shifts of the “slow” and “fast” axis resonances were monitored by the corrections generated by the servo loops and sent to the lasers. The calibration procedure could be implemented in the same manner as for a single sensor, provided a reference FBG is used for strain-to-voltage conversion.

**Figure 11.**
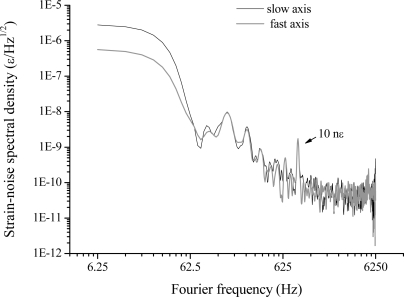
Response to dynamic strain of fast and slow axes cavity modes in laser-locked condition for a 10 nε signal applied to the intra-cavity fiber.

The test experiment on the PM FFP has been carried out only for mechanical deformations so far. Using a low-voltage PZT, attached to the FFP aluminum cylinders, we applied static and dynamic axial strain to the intra-cavity fiber, while the laser and its secondary carrier are frequency locked to two distinct, crossed-polarized resonances. As shown by Figure 11, the sharp peak at 900 Hz demonstrates that the system was capable of detecting smaller signals when compared to a single FBG sensor, thanks to the resonator enhancement effect. Figure 11 shows a strain-noise level approaching 60 pε/√Hz. Sensitivity degradation (by about a factor 10) is expected towards lower frequency, mostly due to ambient noise and laser jitter contributions.

## 3. Acceleration Measurements by FBG Strain Sensors

The monitoring of seismic signals is essential to the study of volcanoes, for surveillance of seismic areas or even in anti-intrusion systems for homeland-security. Activity and movement in the earth is associated with mechanical waves that propagate from the source of the disturbance. These waves, both longitudinal and transverse, cause vibrations that can occur over a large frequency span ranging from quasi-static (below 1 mHz) to acoustic frequencies (above 100 Hz). At present, commercial accelerometers generally operate below 100 Hz. This may leave signals at higher frequencies undetected. Therefore to fully understand the link between the seismic occurrences and seismic signals, detectors with high bandwidth at high sensitivity are required. Velocities and accelerations can be efficiently measured with fiber-optic sensors, provided the mechanical response of the sensor element is known. Telecommunication technology gave great benefits on FBGs, including the provision of low-loss optical fibers and high-quality, relatively-cheap optical components. Fiber-optic accelerometers demonstrate intrinsically-superior sensitivity and small mechanical inertia which overcome typical limitations of conventional seismic accelerometers, making it possible to access a wider acoustic-frequency range. So far, there have been a few examples of fiber-optic accelerometers aimed at such applications. The possibility of strain-to-acceleration transduction was previously demonstrated using a massive flexural beam sensor monitored by a FBG with basic demodulation schemes [22,23].

**Figure 12.**
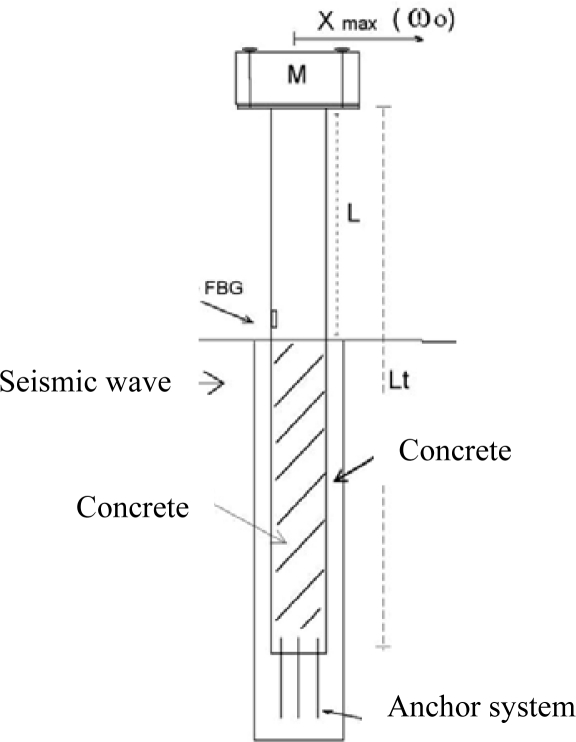
Sketch of the flexural beam acceleration transducer.

Our first approach was a long-beam horizontal accelerometer, containing three different FBGs, which were able to monitor deformations of a rigid cylinder in all directions within the horizontal plane. A large mass (1 kg) was placed on the top of the cylinder while its base was anchored to the ground using a special screw (see Figure 12). Three FBG elements were glued into the cylinder internal surface, parallel to the vertical axis, and placed at angles of 120° apart. Mechanical waves could be detected in the horizontal plane by at least two sensors for determination of their intensity and direction [24].

A customized laser-spectroscopic interrogation technique improves the sensitivity and dynamic response of the system. It relies on two telecom-type DFB diode lasers that track two FBG reflection spectra with high spectral resolution. Since a horizontal seismic wave produces an inertial displacement *x* (*t*) of the mass *m* along the *x* axis in the cylinder section plane, *x* (*t*) can be related to the unknown ground acceleration via a simple mechanical model of the beam, to provide strain to acceleration conversion.

**Figure 13.**
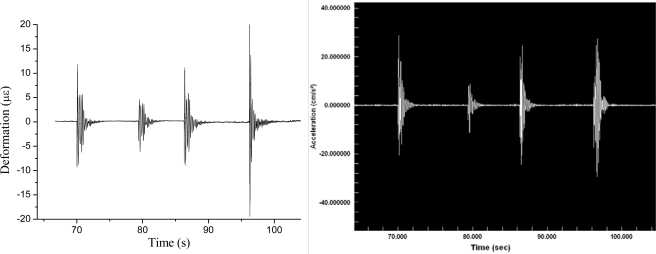
Signals from the two accelerometers with a mechanical pulse train applied to the base. On the left, the strain response of the fiber-optic beam sensor. On the right, the display readout of the K2.

A laboratory test demonstrated successful operation along two directions in the plane for subsequent acceleration pulses. Particularly, in order to evaluate the sensor response to a seismic excitation, we performed a comparison experiment with a commercial K2 (EpiSensor) accelerometer, using a shaking table to apply acceleration to the system in a controlled manner [24]. The two instruments were time synchronized. With the laser tuned near one side of the FBG response curve, a short-pulse sequence was applied to the cylinder sensor along one of the FBG axial directions, which was parallel to horizontal component of the K2 accelerometer. Examples of the output signals from the FBG-sensor and the K2 placed next to it are shown in Figure 13.

More recently, we attached three separate π-shifted FBGs (PSFBGs) to three cantilever beams that flex in orthogonal directions [25]. The PSFBGs present a characteristic response which is quite similar to common optical resonators [26]. The phase defect in the periodic structure indeed modifies its photonic bandgap and creates a sharp resonance exactly at the Bragg wavelength. That strongly improves the capability of detecting small shifts caused by mechanical action on the fiber. The sensors were interrogated by three distributed feedback lasers actively locked to their central resonance using a radio-frequency modulation technique to obtain high sensitivity over a wide dynamic range while preserving a large frequency bandwidth. A cantilever consists of a beam attached to a solid base. It can be considered a simple harmonic system where the end of the beam exhibits a displacement that is proportional to the applied acceleration and the spring constant of the system. The spring constant can be determined by the Young’s modulus of the material, *E*, the beam’s moment of inertia, *I*, and the length of the beam, *L* using the expression

(1) $$k=\sqrt{\mathrm{EI}/L}.$$

**Figure 14.**
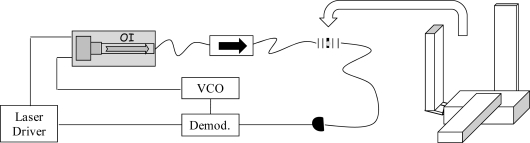
Schematic diagram of the accelerometer’s head. Stainless steel cantilevers are clamped together using aluminum plates. All cantilevers have the same dimensions and nominal resonant frequencies of about 1.5 kHz.

**Figure 15.**
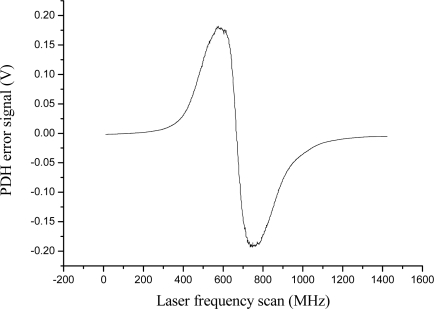
Pound-Drever-Hall error signal obtained by 60 MHz demodulation of the reflected field from a PS FBG.

In our experiment, each cantilever was made of a stainless steal beam (*E* = 200 GPa) 25 mm long, 1 mm wide and 0.1 mm thick. A sketch is depicted in Figure 14. This gives the system a resonant frequency of 1,500 Hz, according to Equation 1. The acceleration of each cantilever could be measured with a PS FBG sensor attached on one side of the beam. The three sensors were all fabricated in SMF-28 fiber with comparable envelope reflectivity (>99%) and resonance linewidth (typically ∼2 pm), but centered at well separated wavelengths: 1,560, 1,540 and 1,578 nm. For this purpose, three separate distributed-feedback (DFB) diode lasers are locked to PSFBGs at the corresponding wavelengths using the Pound-Drever-Hall (PDH) technique. An error signal is generated via sideband modulation of the lasers at 60 MHz and phase-sensitive detection of the PS FBG reflection (an example is shown in Figure 15). The error signal is integrated and fed back to the lasers for locking with a bandwidth of 25 kHz and a dynamic range of approximately 50 g (g = 9.80665 m/s^2^). The feedback signal was recorded to measure the frequency excursions of the FBG and thus the acceleration.

**Figure 16.**
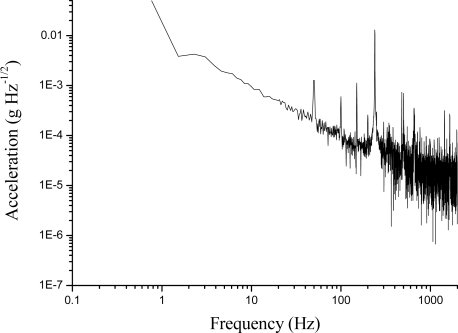
The acceleration noise spectral density along one axis of the accelerometer. A known deformation is applied by a PZT attached to the accelerometer and aligned with the measurement direction to enable the conversion of the sensor’s voltage signal into acceleration.

The acceleration frequency spectrum, obtained by the FFT of the correction signal along one of the three axes, is shown in Figure 16. The system reaches a sensitivity noise floor ranging between 10 and 900 μg/√Hz, in the 10–1000 Hz interval, with similar performance along different orthogonal directions. As expected, at low frequencies the acceleration sensitivity is reduced by the characteristic noise roll-up when approaching DC, due to seismic noise and laser-frequency noise [27]. Indeed, jitter and drifts in laser’s emission frequency represent the main constraint to the minimum detectable deformation [28]. The detection limit can be improved either employing a different laser or decreasing its free-running frequency noise by pre-stabilization onto an external cavity (e.g., a fiber ring resonator).

## 4. Application of Fiber Optic Sensing to Recordings of Musical Instruments

As was shown above, single FBGs, and especially cavities made of two identical FBGs, can be fashioned into very sensitive sensors for strain and vibration. One recently developed application lies in the monitoring of the sound generated by a musical instrument [29]. All “acoustic” string instruments, such as guitars, violins, and even cembalos or harps, have a soundboard that amplifies the sound generated by the string movement. The exact shape of the soundboard determines the coloration of the instrument through, e.g., the presence of harmonic excitations and vibrational nodes. Most serious musicians prefer to record the sound of their instrument using a high quality microphone, but this may be impractical in an environment with large background “noise” such as a performance stage. Many acoustic guitars and, to a much lesser extent, other string instruments are therefore equipped with piezoelectric transducers (PZT “pick-ups”) that are placed on the soundboard and convert its vibrations into an electrical signal. While pick-ups have a fairly flat frequency response between about 100 Hz–20 kHz and are inexpensive, they also have comparably high inertia, and are difficult to amplify due to their high impedance. When many of these PZTs are mounted onto a single instrument, the vibrations of the soundboard maybe affected and the instrument coloration may be altered.

Fiber optic transducers are preferred when size and inertial mass is a concern. In the following sections, two sensors are briefly described, one based on the acoustic modulation of the reflectivity of a single FBG near its mid-reflection point and another one based on the reflectance near a cavity fringe of a cavity made of two identical FBGs.

### 4.1. Single FBG Transducer

In a preliminary study we have demonstrated the feasibility of acoustic transduction by affixing a single FBG on a guitar body and comparing the recordings with those made by a PZT [29]. A DFB laser (AC 5900, Archcom Technologies) was set to a wavelength near the mid-reflection point of a commercial FBG (reflection bandwidth: 0.2 nm; peak attenuation 30 dB) and the transmitted light was monitored by a fast InGaAs photodiode (DET10C, Thorlabs). Recordings were made at a sampling rate of 44.1 kHz when individual strings were plucked. The Fourier transform of these traces showed very similar frequency response for both the PZT and the FBG transducers as expected. Differences could be readily attributed to the difference in positions at which the sensors are placed on the soundboard, *i.e.,* under the bridge (PZT) and about half distance between bridge and guitar rim (FBG). Graphical representations are not very informative regarding the sensors’ performance and we refer to the electronic material associated with reference [29] for audio recordings.

### 4.2. FBG Cavity Transducer

**Figure 17.**
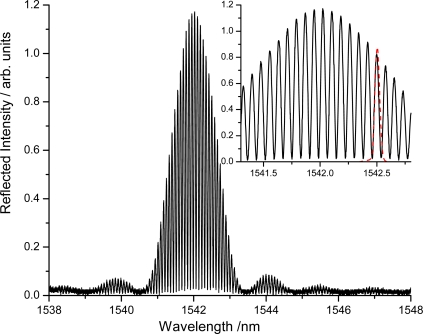
Reflection spectrum of an optical cavity made from two low reflectance FBGs spaced by 10 mm. The insert shows the laser emission spectrum as a red dashed line.

**Figure 18.**
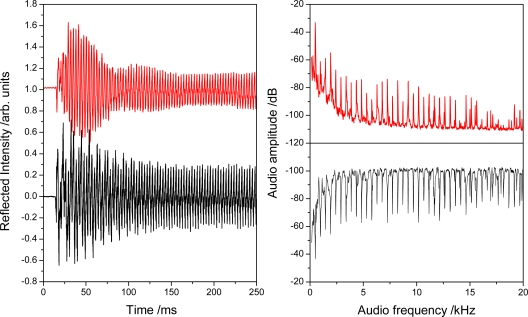
Left: Response of the FBG transducer (top) and the PZT to a plucked E4 string. Right: The Fourier transform of the respective complete waveforms shows the fundamental frequency at 326.8 Hz and overtones up to 12 kHz.

More recently we used a Fabry-Perot cavity made of two identical FBGs that were placed 10 mm apart to obtain sound recordings at higher sensitivity and lower noise. The FBG cavity (QPS Photronics, Montreal, QC) was deliberately made to have a very low finesse (F < 5) as is clearly apparent from the inset of the cavity reflection spectrum (Figure 17). A DFB laser (QPS Photronics) was current (temperature) tunable and was periodically reset to a wavelength near the mid-reflection point of a cavity fringe. Light from the laser was directed to the FBG cavity which was fixed onto the soundboard of the guitar with adhesive tape. The reflected light was directed by a fiber optic circulator into a photodetector. The photodiode signal was amplified and sampled using an audio preamplifier (Edirol UA-25EX). Figure 18 shows the first 250 ms of a 10 s time evolution of the transmitted light signal and the PZT response as the guitar’s E_4_ string is plucked. The Fourier transforms of the entire PZT and FBG transducer recordings show the fundamental frequency of the E_4_ note (here slightly mistuned to 326.8 Hz) as well as its harmonics up to the 25th overtone at 8,171 Hz. Both transducers could reproduce frequencies higher up to 22 kHz with the FBG transducer exhibiting a lower noise floor. The difference in their frequency response is partly due to the different position on the guitar body.

Recordings with other musical instruments were made and, of those, the solid body electric guitar and solid body electric bass guitar were particularly promising. Recordings made with a violin and a different louder acoustic guitar showed evidence of clipping of the higher vibrational modes. Instruments in which the sound is generated by a resonating air column (woodwinds, brass instruments) also showed a response, but the sensitivity to high frequencies was poor.

Future work will focus on improving further the sound quality by using a Pound-Drever-Hall feedback scheme similar to that described in section 3 [7].

## 5. Chemical Sensing by Optical-Fiber Ring Resonators

Chemical sensors using fiber-optic methodology are the subject of extensive research and development activity with potential applications in industrial, environmental and biomedical monitoring [30–32]. In this context, a miniature chemical sensor combining laser spectroscopy and state-of-the-art optical fiber technology may be suitable to in-situ, non-invasive gas and liquid analysis with high selectivity and sensitivity. This can be based on either direct or indirect (indicator-based) detection techniques [33,34]. In the former scheme, the optical properties of the analyte, such as refractive index (RI), absorption or emission, are measured directly. In the indirect scheme, the color or fluorescence of an immobilized label compound, or any other optically-detectable bioprocess, are monitored. In recent years, interrogation techniques have further advanced with the use of spatially-resolved spectroscopy [35], evanescent-wave spectroscopy [36–38] as well as surface-plasmon resonance [39–42]. Sensors have also been incorporated into passive optical cavities consisting of fiber loops or linear fiber cavities defined e.g., by two identical FBGs [43–45]. These cavities have shown to be effective means of amplifying the sensors response. Their application to mechanical sensing is reviewed elsewhere in this issue. Optical microresonators, of different geometries, have been also used as label-free and ultrasensitive chemical sensors over the past several years [46–49]. In all cases above, a change in ambient refractive index may lead to a wavelength shift of the cavity modes, if part of the evanescent wave of the mode is exposed to the environment. On the other hand, if the molecules exhibit absorption lines or bands in the vicinity of the resonance wavelength, the cavity lifetime, namely the ring-down time (RDT), will be reduced, leading also to a reduction in power transmitted through the resonator and in the quality (Q-) factor.

In our research, a passive optical-fiber ring (OFR) resonator has been developed for chemical analysis of liquids. As is well known, a light leakage from the fiber changes the resonator finesse. In our experiment, along a short region of the fiber within the loop, the core is exposed to a liquid sample containing the species of interest. The presence of this analyte can be measured, for example, by monitoring the light loss due to optical absorption or the refractive index changes through evanescent-wave interaction. Preliminary results on operation and test are presented.

The reflected and transmitted intensities from the cavity can be expressed from the multiple interference of superimposed signals circulating in the fiber ring. The round-trip phase-lag *φ* in such a cavity is given by $\phi =\;\frac{4\pi \;n\left(l\right)}{\lambda }$, where *n* is the refractive index of the medium (*i.e.*, fiber core), *l* is the half-length of the loop, and *λ* is the propagating wavelength, while the finesse can be defined as $F=\;\frac{\pi \;\sqrt{k}}{1-k}$ with *k* the coupling coefficient of radiation to the cavity [50]. The observed peak’s full width at half maximum (FWHM) can be characterized by $\delta \nu =\;\frac{\text{FSR}}{F}$, where *FSR* is the free spectral range. The height and width of the peaks change due to additional loss by the chemicals in the sensitive part of the fiber [36].

**Figure 19.**
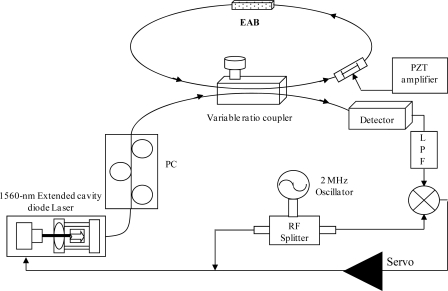
Schematic of the fiber-ring resonator. PC: polarization controller; EAB: evanescent-access block; LPF: low-pass filter.

**Figure 20.**
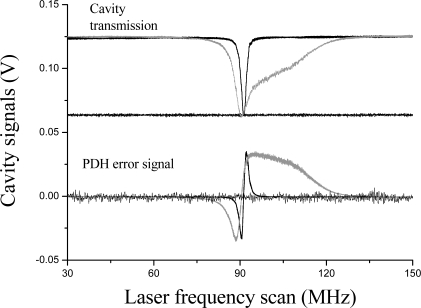
Locking of the laser to the fiber cavity resonance. Top: cavity transmitted power for free-running (black solid line) and locked laser with different low and high servo gains (gray and dotted line, respectively). Bottom: PDH error signal in unlocked (black solid line) and locked cases (gray and dotted line, respectively).

The experimental set-up used to interrogate the fiber resonator and extract the absorption information is shown in Figure 19. The OFR was built using SMF-28 fiber (length is about 2.5 m) and a variable-ratio fiber coupler (1—99%) to inject near-infrared radiation into the cavity. A fiber evanescent-field access block (EAB) allowed the (evanescent) cavity-field to interact with the external environment. We use an extended-cavity diode laser emitting in the telecom region of the spectrum (1,560 ± 30 nm) as a coherent-light source. It was frequency modulated at about 2 MHz through its bias-tee input, so that symmetrical sidebands are superimposed on the carrier frequency. The laser, equipped with a single-mode polarization-maintaining (PM) fiber pig-tail, was directly connected to one port of a single-mode coupler via a fiber polarization controller (PC) in order to define the state of polarization and select a specific cavity eigenmode. The fiber coupler also collected the radiation that partially leaked out from the loop. The transmission peaks observed on cavity resonances over a laser-frequency sweep are shown in Figure 20.

The EAB had an effective length of ≈ 1 mm and was devised to have a 2.6% residual transmission (k = 0.974) with overlay of matching index (1.467). The sensing element of the fiber was placed on a glass substrate on which chemicals could be readily applied. The overall coupling coefficient, which is determined by the variable coupler only if the external refractive index is below 1.467, affects the overall finesse, theoretically ranging from 3 to 300. A piezo-electric transducer was glued on the fiber for modulation of the loop length (stretching range 10 μm in the 0–1 kHz frequency range) when scanning of the loop’s resonances was necessary. The interrogation principle relies on heterodyne detection of the output signal from the OFR when the source is tuned into resonance with it. In particular, the signal coming from the loop was detected by an InGaAs PIN photodiode (Thorlabs DET01CFC) and amplified by a 500 MHz amplifier (MiniCircuit ZFL-500LN). Demodulation at 2 MHz was performed by a double-balanced mixer, generating an error signal for laser locking to the resonance according to the Pound-Drever-Hall technique (see section 2.2). The PDH signal was sent to a proportional-integrative circuit (servo) that provided the necessary feedback to the laser. In this way, the laser could be frequency-stabilized to a cavity mode for several hours, without suffering from thermal fluctuations of the environment. Locking action is shown in Figure 20.

**Figure 21.**
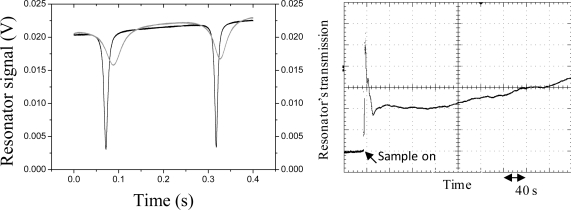
Cavity transmission signals in different cases. Left: free-running laser case with no sample on the EAB (black line) and with a sample (glycerol diluted at 99.5% with D_2_O) causing a small index overlay (gray line); right: laser-locked condition with the sample on the EAB.

Our first test of the sensor’s response consisted of covering the EAB with a solution containing glycerol diluted by 5% D_2_O, which does not absorb strongly around 1,560 nm (compared to H_2_O). In this way, the external index increased nearly to the critical value and the light intensity dropped dramatically in typically few seconds. The cavity response (transmission and error signal) was stored before and after applying the sample. The EAB could be cleaned by ethanol to restore the initial condition. When the sample was applied to the EAB, the cavity resonances (Figure 21, left) remained symmetric while the width of the signal was increased as expected from finesse degradation. Hence a fraction of the guided core mode leaked out from the cavity due to the high-index glycerol cladding. The same has been obtained when the laser was locked to the cavity mode (Figure 21, right), and the continuous transmission trace showed a sudden signal reduction with application of the sample (bottom-right), with a further slower decrease due to the evaporation of water with time. This effect was particularly evident because the external index was much closer to the maximal value permissible for light confinement. Nevertheless, the laser remained always locked to the cavity. As the experiment is repeated with increasing concentration of heavy water (10%, 20%, 30%, 50% in glycerol) the refractive index decreases and with it the cavity loss.

A particular liquid species, with a strong absorption band around the laser wavelength, can thus be diluted in a glycerol-water “index-matching” solution and the absorption-induced loss on the transmission detected as a function of wavelength and concentration, similarly to direct absorption spectroscopy. At present, work is in progress to identify and quantify different species in a sample measuring the cavity ring-down time (RDT) that carries the information on the absorbance. The RDT will be extracted by resonant coupling of an intensity-modulated light beam into the cavity and measuring the modulation frequency-dependent phase-shift of light exiting the resonator by a lock-in amplifier. This approach is referred to as the phase-shift cavity ring-down (PS-CRD) and can be applied to fast and sensitive retrieval of the chemically-induced resonator losses [9,36]. An improved version comprising a widely-tunable laser and an EAB with longer exposed length is underway.

## 6. Conclusions

We have developed several experimental systems devoted to interrogation of optical fiber sensors, which are strongly based on the extension of laser-spectroscopic methods to analyze their reflected radiation. They demonstrated a great potential in terms of sensitivity and precision for different fields, such as geophysical survey, structural health monitoring, accelerometers and chemical sensing. For most sensors, active frequency locking of lasers to the sensors revealed to be very effective for real-time, low-noise monitoring. Particularly, the use of optical fiber resonators provided a superior sensitivity level in measurements of deformation and temperature without adding significant complexity. This is the case of FBG resonators for 10^−12^ static and dynamic strain detection. On the other hand, the use of highly-birefringent fibers represents a possible solution to thermal cross-sensitivity. Some relevant applications are also shown, including wide-bandwidth accelerometers and acoustic instrument tools. Finally, a preliminary evidence of evanescent-wave sensing in liquids by an optical fiber-ring resonator is provided.
